# Population genetic structure of the endemic rosewoods *Dalbergia cochinchinensis* and *D. oliveri* at a regional scale reflects the Indochinese landscape and life‐history traits

**DOI:** 10.1002/ece3.3626

**Published:** 2017-12-01

**Authors:** Ida Hartvig, Thea So, Suchitra Changtragoon, Hoa Thi Tran, Somsanith Bouamanivong, Ida Theilade, Erik Dahl Kjær, Lene Rostgaard Nielsen

**Affiliations:** ^1^ Department of Geosciences and Natural Resource Management University of Copenhagen Frederiksberg C Denmark; ^2^ Institute of Forest and Wildlife Research and Development, Forestry Administration Ministry of Agriculture, Forestry and Fisheries Phnom Penh Cambodia; ^3^ Forest and Plant Conservation Research Office Department of National Parks, Wildlife and Plant Conservation Ministry of Natural Resources and Environment Chatuchak, Bangkok Thailand; ^4^ Forest Genetics and Conservation Center for Biodiversity and Biosafety Institute of Agricultural Genetics Vietnam Academy of Agricultural Sciences Hanoi Vietnam; ^5^ National Herbarium of Laos Biotechnology and Ecology Institute Ministry of Science and Technology Vientiane Laos; ^6^ Department of Food and Resource Economics University of Copenhagen Frederiksberg C Denmark

**Keywords:** forest cover change, Indochina, landscape genetics, Mekong River, plant mating systems, tropical trees

## Abstract

Indochina is a biodiversity hot spot and harbors a high number of endemic species, most of which are poorly studied. This study explores the genetic structure and reproductive system of the threatened endemic timber species *Dalbergia cochinchinensis* and *Dalbergia oliveri* using microsatellite data from populations across Indochina and relates it to landscape characteristics and life‐history traits. We found that the major water bodies in the region, Mekong and Tonle Sap, represented barriers to gene flow and that higher levels of genetic diversity were found in populations in the center of the distribution area, particularly in Cambodia. We suggest that this pattern is ancient, reflecting the demographic history of the species and possible location of refugia during earlier time periods with limited forest cover, which was supported by signs of old genetic bottlenecks. The *D. oliveri* populations had generally high levels of genetic diversity (mean *H*_e_ = 0.73), but also strong genetic differentiation among populations (global *G*_ST_ = 0.13), while *D. cochinchinensis* had a moderate level of genetic diversity (mean *H*_e_ = 0.55), and an even stronger level of differentiation (global *G*_ST_ = 0.25). These differences in genetic structure can be accounted for by a higher level of gene flow in *D. oliveri* due to a higher dispersal capacity, but also by the broader distribution area for *D. oliveri*, and the pioneer characteristics of *D. cochinchinensis*. This study represents the first detailed analysis of landscape genetics for tree species in Indochina, and the found patterns might be common for other species with similar ecology.

## 1 INTRODUCTION

The region of Indochina, the mainland part of Southeast Asia, is recognized as part of one of the world's biodiversity hot spots. The area harbors a high overall biodiversity and very high levels of endemism for both plant and animal species, but is also experiencing high rates of primary habitat loss and currently has one of the world′s highest levels of deforestation (Myers, Mittermeier, Mittermeier, da Fonseca, & Kent, 2000; WRI 2015).

The high species richness and endemism levels in Southeast Asia can, in part, be attributed to the complex biogeographical and geological history of the region (Woodruff, 2010), involving collisions of the Indian and Australian tectonic plates early in the Cenozoic, followed by subsequent movements of these and other smaller plates, which caused rapid changes in the distribution of land and sea areas in the region (Hall, 2001). In more recent times, the region has experienced even more extreme fluctuations in sea levels due to temperature changes in glacial and interglacial periods in the Quaternary (Hall, 2001). These changes were followed by associated changes in vegetation types and cover, although the specific distribution of vegetation types during glacial and interglacial periods in the region remains debated (Bird, Taylor, & Hunt, 2005; Heaney, 1991; Woodruff, 2010; Wurster et al., 2010). The highly topographically varied landscape of Indochina also contributes to its rich biodiversity. The region contains both mountainous regions and lowland areas, as well as the large freshwater bodies the Mekong River and the Tonle Sap Lake, and is thus supporting a diverse range of habitats from forests and grasslands to freshwater and coastal habitats (Maxwell, 2004; Theilade, Schmidt, Chhang, & McDonald, 2011; Tordoff et al., 2012).

Large parts of Indochina have been inaccessible to researchers for long periods during the 20th century due to political instability and lack of infrastructure, and the biodiversity is in many areas severely underexplored (Theilade & de Kok, 2015; Tordoff et al., 2012). In recent years, the general accessibility of more remote areas, especially in Cambodia, has improved along with the general development in the region. High numbers of new plant and animal species have been described in the region recently, and many more probably remain to be discovered (Giam, Ng, Yap, & Tan, 2010; WWF 2014).

However, often very little is known about the distribution, population dynamics, and life‐history traits of single species, and how the genetic diversity of species and populations is affected by the Indochinese landscape as well as the current habitat changes in terms of deforestation and fragmentation. Besides the direct negative effect of habitat loss, species are also affected by the associated genetic impacts following reduced population size and fragmentation, such as increased levels of inbreeding, genetic drift, and loss of genetic diversity (Lowe, Harris, & Ashton, 2004). Assessment of the diversity and structure of intraspecific genetic variation as well as acquiring basic knowledge on traits such as reproduction and dispersal systems can contribute toward a more general understanding of how landscape and habitat changes affect species and populations. Such knowledge is also important in order to guide conservation and management of remaining populations of threatened species. The majority of studies exploring phylogeographic patterns and population divergence in the region has either focused on the divergence between mainland and island SE Asia (Ohtani et al., 2013; Patou et al., 2010) or the northern part extending into Southern China (Yu & Nason, 2013). The population structure of forest‐associated species within central Indochina is less well studied (Blair et al., 2013; Fuchs, Ericson, & Pasquet, 2008; Morgan et al., 2011), and particularly Cambodia is underrepresented in the sampling. A few studies assess population structure of plants or trees in the region although most are limited to the local scale (Pakkad, Kanetani, & Elliot, 2014; Senakun, Changtragoon, Pramual, & Prathepha, 2011; Wattanakulpakin, Iamtham, Grubbs, & Volkaert, 2015; Zhao & Zhang, 2015; Zhao et al., 2013).

**Figure 1:**
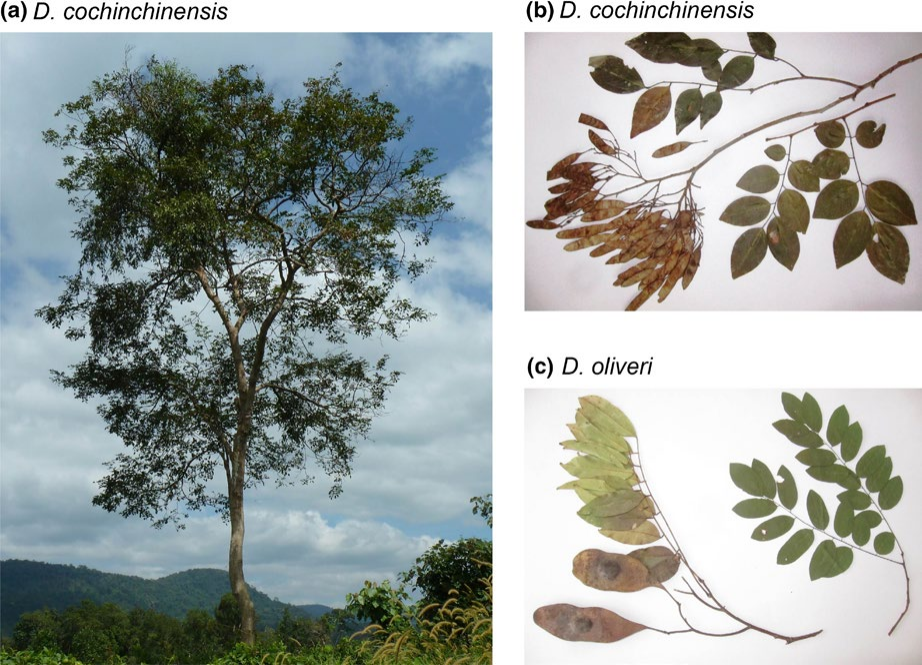
Study species. (a) Habit of *Dalbergia cochinchinensis*, Koh Kong, Cambodia, (b) leaves and fruits of *D. cochinchinensis,* and (c) leaves and fruits of *Dalbergia oliveri*. Photographs: I. Hartvig

This study explores the genetic structure of two endemic tree species of the genus *Dalbergia* L.f. (Fabaceae: Papilionoideae) across the Indochinese landscape. *Dalbergia cochinchinensis* Pierre and *Dalbergia oliveri* Gamble ex Prain (Figure 1) are two of the most valuable timber species in Indochina and are highly sought after for their attractive wood, which is used especially for luxury furniture (CTSP 2004; GW 2015; Niyomdham, Hô, Dy Phon, & Vidal, 1997). Both species occur in Thailand, Laos, Cambodia, and Vietnam and are often found in the same localities, although *D. oliveri* has a wider distribution area also extending into Myanmar and occurs in a broader range of forest types than *D. cochinchinensis* (CTSP 2004; Niyomdham, 2002; Niyomdham et al., 1997). Until recently, there was some confusion concerning the taxonomy of *D. oliveri*, as it was earlier divided into several species and, for example, was known as *D. bariensis* in Cambodia and *D. oliveri* in Vietnam. Recent DNA barcoding analysis, however, confirmed that *D. oliveri* constitutes a single, widespread species (Hartvig, Czako, Kjaer, & Nielsen, 2015), a result which is consistent with the most recent morphological revision by Niyomdham et al., 1997.

Overexploitation as well as habitat loss has led to dramatic decreases in their overall population size (CTSP 2004; EIA 2015), and both species are listed as threatened on the IUCN redlist (Nhgia, 1998a,b) as well as included in App. II of the Convention on International Trade in Endangered Species (CITES) in order to prevent international trade of illegally logged timber (CITES 2017). Despite their economic and ecological significance, previous population‐level studies of these two species are limited and cover only small parts of their distribution areas (Moritsuka, Tagane, Toyama, Yahara, & Tachida, 2013; Phong, Hien, Thanh, & Tang, 2011; Soonhua, Piewluang, & Boyle, 1994; Yooyuen, Duangjai, & Changtragoon, 2011).

Here, we explore and compare the wide‐scale population genetic structure of *D. cochinchinensis* and *D. oliveri* and relate the discovered patterns to landscape characteristics as well as life‐history traits and reproductive strategies of the two species.

By selecting two species, which distributions are limited to specific habitats and still span most of the Indochina region, we aim to detect common trends in population genetic structure that could be caused by past and/or present landscape patterns and changes in habitat cover. On the other hand, we can observe to which extent differences between the two species can be attributed differences in life‐history traits.

As both species are endangered with continually decreasing population sizes, the results can also be directly used to inform and guide management authorities when designing conservation plans.

For these purposes, we apply highly variable microsatellite markers specifically developed for this study to samples of *D. cochinchinensis* and *D. oliveri* from natural populations in Laos, Thailand, Cambodia, and Vietnam. The sampling spans the entire distribution range for *D. cochinchinensis* and the majority of that for *D. oliveri*. Our specific objectives are to 1) explore and describe the landscape genetic diversity patterns of the two species across the study area, 2) relate the patterns to current and ancient geographical characteristics of the Indochina landscape, 3) evaluate whether the recent deforestation and fragmentation levels have impacted the genetic diversity and inbreeding level of the two species, and 4) compare the genetic structure between the two species and relate it to differences in life‐history traits.

## 2 MATERIALS AND METHODS

### 2.1 Study species

*Dalbergia cochinchinensis* and *Dalbergia oliveri* are medium‐large trees reaching up to 35 m in height and a diameter at breast height (DBH) up to 90 cm. Both species produce attractive red colored wood, which is durable and resistant to termites (CTSP 2004). Although similar in habitus, the two species are easily distinguished by leaf and bark morphology, as well as flower color, which is white for *D. cochinchinensis* and light purple for *D. oliveri*. Flowers are small and fragrant, and both species are thought to be pollinated by small insects. The pods are dry with only few seeds, and while they are thin and papery in *D. cochinchinensis*,* D. oliveri* has larger and more leathery pods with thick walls around the hollow seed spaces (Niyomdham et al., 1997). Wind and/or water dispersal seem likely for these species. Phylogenetic analyses do not suggest a close relationship between *D. cochinchinensis* and *D. oliveri* (Hartvig et al., 2015; Vatanparast et al., 2013), and there is no knowledge of hybridization between them.

*Dalbergia cochinchinensis* occurs in deciduous and semi‐deciduous forests at altitudes ranging from 0 to 1200 m and can grow on most soil types (CTSP 2004). It is found in protected areas in central, eastern, and northeastern Thailand and central and southern Vietnam. In Cambodia, it is found in many provinces and is especially frequent in the northeast regions along the Thai border (CoP16 C, 2013; CTSP 2004; Niyomdham, 2002). It is registered in a few provinces in central and southern Laos (CoP16 C, 2013; van Sam, Nanthavong, & Kessler, 2004), but the distribution in this country seems less studied. *Dalbergia cochinchinensis* is recognized as an intermediate pioneer species (So, 2000), with high growth rates in young stages and lower in older stages, and it has a strong ability of coppice regeneration (CoP16 C, 2013).

*Dalbergia oliveri* occurs in dry deciduous, semi‐deciduous, and evergreen forests usually at altitudes below 1000 m and often in moist areas, along streams and rivers and on hill sides (Aerts et al., 2009; CTSP 2004). It is found throughout many provinces in Cambodia and in protected areas in southern Vietnam (CTSP 2004), and in Thailand, it is known as common in the north, northeast, and eastern regions. It is reported from a few provinces in Laos and Myanmar (van Sam et al., 2004; USNH 2003), but the distribution in these countries is not well known. Growth rate is reported as slow and natural regeneration poor, but coppice regeneration is very strong (CTSP 2004 and pers. obs.).

We sampled 677 individuals from 26 populations of *D. cochinchinensis* from localities in Laos, Thailand, Cambodia, and Vietnam and 616 individuals from 23 populations of *D. oliveri* from localities in Laos, Cambodia, and Vietnam in the period from November 2010 to October 2014 (Table 1, Fig. S1). Eight of the localities ov erlapped between the two species (Tables S1 and S2, Fig. S1). We aimed at sampling mature individuals min. 10 m apart, however, as populations varied greatly in size, density, levels of logging, and accessibility, this protocol could not be followed strictly in all populations. Leaf or cambium samples were collected from each individual and dried immediately in silica gel, and DBH (diameter at breast height) was measured (with few exceptions). For estimation of outcrossing rate, we collected mature pods from two populations of *D. cochinchinensis* (NOY and SPEU) and one population of *D. oliveri* (RBL). A total of 711 seeds from 17 mother trees from the NOY population, 382 seeds from 14 mother trees from the SPEU population, and 381 seeds from 18 mother trees from the RBL population were sown without any prior treatment in an app. 50/50 mix of vermiculite and standard potting soil and kept in a glasshouse at min. 25°C and watered daily. Leaves from seedlings were collected and stored at −20°C until DNA extraction.

**Table 1:** Genetic characteristics of *Dalbergia cochinchinensis* and *Dalbergia oliveri* populations

Country	Pop code	*R*	*N*	*Na*	*Na*(rare)	Priv. all.	*H*_*o*_	*H*_e_	*F*_IS_	*T*_2_	*M*_m_
*D. cochinchinensis*											
Laos	NIA	0.83	21	2.67	2.20	0	0.39	0.43	0.078	1.167	0.37
	TXA	0.80	13	2.22	1.96	0	0.36	0.34	−0.029	0.750	0.45
	TAL	0.76	17	2.56	2.14	0	0.37	0.40	0.089	0.910	0.48
	BAN	1.00	28	3.00	2.23	0	0.45	0.43	−0.076	0.796	0.43
	TAI	0.71	18	3.11	2.33	0	0.46	0.43	−0.041	0.215	0.41
	UDO	1.00	28	3.33	2.36	1	0.40	0.43	0.086	0.604	0.41
	KPN	0.72	19	3.33	2.28	0	0.45	0.39	−0.115	−1.252	0.39
Thailand	PW	1.00	30	4.56	2.61	1	0.43	0.48	0.085	−2.006	0.32
	PK	1.00	29	3.67	2.50	0	0.41	0.48	0.140	0.314	0.42
	PY	1.00	33	4.56	2.64	2	0.45	0.48	0.030	−1.488	0.33
	PJ	1.00	22	7.22	3.78	3	0.68	0.67	0.000	−2.420	0.31
Cambodia	POS	0.93	28	7.33	4.15	2	0.79	0.75	−0.024	0.569	0.32
	RUK	0.44	15	5.44	3.75	0	0.69	0.70	0.027	1.196	0.32
	ANG	0.16	4	3.22	3.22	0	0.72	0.62	−0.026	a	0.24
	NOY	0.96	26	5.56	3.52	1	0.65	0.66	0.029	1.015	0.28
	PYA	0.84	27	6.89	4.01	2	0.72	0.71	0.017	0.332	0.36
	RO	0.42	11	6.22	3.50	1	0.72	0.66	−0.040	−0.943	0.28
	KRA	0.46	14	5.00	3.53	0	0.69	0.62	−0.071	0.177	0.30
	SK	0.79	24	5.89	3.48	1	0.68	0.64	−0.046	−0.278	0.33
	DP	0.33	10	4.44	3.58	1	0.68	0.61	−0.057	0.912	0.31
	KIR	0.86	25	6.00	3.58	1	0.66	0.64	−0.036	−0.465	0.34
	SPEU	0.78	22	5.67	3.05	0	0.70	0.65	−0.051	0.745	0.33
	DNT	0.56	6	3.44	3.38	2	0.44	0.46	0.102	a	0.30
	HAP	1.00	25	5.44	3.97	2	0.63	0.62	0.015	0.746	0.38
Vietnam	CAH	1.00	6	3.11	2.73	0	0.53	0.47	−0.061	a	0.36
	YOD	0.53	22	3.89	3.28	1	0.47	0.50	0.079	0.748	0.36
Overall	Mean	0.76	20	4.53	3.95	‐	0.56	0.55	0.004	‐	0.35
*D. oliveri*											
Laos	NIA	0.80	9	5.86	4.64	1	0.83	0.73	−0.075	a	0.31
	TXA	0.75	13	7.14	5.23	0	0.81	0.79	0.012	1.621	0.34
	BAN	1.00	5	4.86	4.86	0	0.89	0.72	−0.122	a	0.32
	TKK	0.91	22	6.86	4.21	4	0.67	0.68	0.035	−0.379	0.31
Cambodia	SMK	0.97	32	9.00	5.21	0	0.83	0.80	−0.019	1.066	0.32
	TBM	0.97	38	9.71	5.22	0	0.83	0.80	−0.032	1.068	0.40
	PL	0.63	6	6.00	5.68	0	0.93	0.79	−0.108	a	0.27
	RO	0.25	8	6.86	5.55	1	0.80	0.77	0.025	a	0.32
	KRA	0.56	16	10.29	5.99	0	0.81	0.82	0.047	0.081	0.39
	DP	0.17	6	5.43	5.12	0	0.93	0.77	−0.114	a	0.29
	KIR	0.48	15	8.29	5.36	0	0.85	0.78	−0.057	0.467	0.37
	DNT	0.36	9	7.00	5.50	1	0.76	0.78	0.087	a	0.31
	RBL	0.83	39	9.86	5.30	1	0.78	0.80	0.044	1.333	0.33
	LUM	0.94	31	9.57	5.21	0	0.82	0.79	−0.016	0.175	0.33
	SEI	0.83	30	5.86	3.82	0	0.77	0.67	−0.142	1.171	0.37
Vietnam	CMR	1.00	33	9.86	5.07	2	0.71	0.76	0.074	−2.721	0.36
	YOD	0.91	21	10.71	5.90	4	0.68	0.82	0.062	−0.948	0.34
	CYS	0.56	32	7.29	4.29	0	0.69	0.66	−0.118	−1.175	0.32
	LUS	0.91	21	5.14	3.34	0	0.68	0.61	−0.092	−0.033	0.23
	DAL	1.00	24	5.14	3.99	0	0.68	0.61	−0.045	−0.702	0.29
	CTA	1.00	25	6.57	4.11	3	0.65	0.64	−0.004	−0.205	0.35
	CTB	0.42	6	3.71	3.52	0	0.55	0.58	0.142	a	0.29
	SAM	0.96	24	6.43	3.75	1	0.63	0.64	0.022	−1.201	0.29
Overall	Mean	0.75	20	7.34	4.82	‐	0.76	0.73	−0.017	‐	0.32

*R* = clonal richness estimate, *N *= number of unique genotypes used for further analyses, *Na* = mean number of alleles, *Na*(rare)  = mean number of alleles corrected for sample size differences, Priv. all.= number of private alleles, *H*_*o*_=observed heterozygosity, *H*_e_ = expected heterozygosity, *F*_IS_ = Fixation index, *T*_2_ = bottleneck statistic representing deviation between observed and expected heterozygosity based on number of alleles, *M*_m_ = Garza–Williamson index representing ratio between number of alleles and allelic range (See Materials and Methods for further details).

a Sample size too low for accurate estimation of this value.

### 2.2 DNA extraction and genotyping

DNA from leaf or cambium samples was extracted using a CTAB protocol (Doyle & Doyle, 1987) or (for seedlings) the PowerPlant^®^ Pro DNA isolation kit (MO BIO Laboratories, Inc., CA, USA).

Microsatellite markers for *D. cochinchinensis* and *D. oliveri* were developed by Genoscreen (Lille, France), using enriched genomic libraries and pyrosequencing as described by Malausa et al. (2011). For each species, libraries were constructed using pooled genomic DNA from 10 samples, and 12 microsatellite markers identified from the sequences were then validated on a panel of 30 samples. Library construction and validation of markers were based on Cambodian samples for *D. cochinchinensis,* and Cambodian and Vietnamese samples for *D. oliveri*. Nine (COC_01, COC_05, COC_06, COC_07, COC_08, COC_10, COC_11, COC_13, COC_18) and eight (OLI_01, OLI_05, OLI_06, OLI_14, OLI_15, OLI_16, OLI_17, OLI_19) polymorphic markers with high amplification rates were selected for *D. cochinchinensis* and *D. oliveri*, respectively, for use in this study (characteristics are given in Tables S3 and S4). The markers were multiplexed into two mixes for each species (*D. cochinchinensis* MIX1: COC_01, COC_05, COC_10, COC_13, and MIX2: COC_06, COC_07, COC‐08, COC_11, COC_18, and *D. oliveri* MIX1: OLI_01, OLI_05, OLI_06, OLI_16, and MIX2: OLI_14, OLI_15, OLI_17, OLI_19), and PCRs were performed using the QIAGEN Multiplex PCR kit (QIAGEN, Sweden) according to the manufacturer's instructions, except that total reaction volume was reduced to 10 μl. The PCRs were conducted on a Bio‐RAD C1000 Thermal Cycler (Bio‐Rad Laboratories, Denmark) or a GeneAmp 2700 Thermal Cycler (Applied Biosystems, USA) with the following cycling conditions: 95°C for 15 min, followed by 30 cycles of 95°C for 30 s, 55°C for 90 sec, and 72°C for 90 s, and a final step at 72°C for 10 min.

Fragment sizes were determined on an ABI 3130XL Genetic Analyzer (Applied Biosystems, USA) and analyzed using the microsatellite plugin in Geneious 6.0.5 to 7.1.7 (Biomatters Inc., USA).

### 2.3 Genetic data analysis

#### 2.3.1 Genetic diversity

The marker OLI_1 showed poor amplification in some populations, and the total percentage of missing data for this locus across all samples was 18%. This locus was therefore removed from the *D. oliveri* dataset, before any analyses.

An initial screening of the samples showed many redundant genotypes for both species. We therefore used GenAlEx 6.501 (Peakall & Smouse, 2006, 2012) to conduct a Probability of Identity analysis for each population separately in order to test whether identical genotypes could be considered as clones. To provide an estimate of clonal versus sexual reproduction in each population, a clonal richness estimate, $R=\;\left(G-1\right)/\left(N-1\right),$ where *G* is the number of unique genotypes and *N* is the number of samples, was calculated according to Dorken and Eckert (2001).

Duplicate genotypes were removed before any subsequent analysis (to 523 genotypes for *D. cochinchinensis* and 465 genotypes for *D. oliveri*).

We first tested the datasets for deviations from Hardy–Weinberg (HW) proportions using a modified Markov chain algorithm as described in Guo and Thompson (1992), as well as for linkage disequilibrium (LD) between pairs of microsatellite loci using a likelihood ratio test (Excoffier & Slatkin, 1998). Both tests were performed in Arlequin Ver. 3.5.2 (Excoffier & Lischer, 2010) and the results evaluated using a 5% table‐wide significance level. MICRO‐CHECKER (Van Oosterhout, Hutchinson, Wills, & Shipley, 2004) was used to test the datasets for null alleles and genotyping errors.

We also estimated the frequency of null alleles with the software FreeNA (Chapuis & Estoup, 2007), which computes a dataset with corrected estimates of the alleles frequencies. *F*_ST_ (Weir & Cockerham, 1984) values for both the original and the corrected dataset were then calculated and compared in order to evaluate the effect of null alleles in the dataset.

We used GenAlEx to calculate the following measures of population genetic diversity (for all loci): mean number of alleles (*N*_a_), number of private alleles (*N*_p_), observed heterozygosity (*H*_o_), and expected heterozygosity (*H*_e_). The number of observed alleles corrected for sample size differences, *Na* (rare), was calculated in HP‐rare 1.1 (Kalinowski, 2005), and population size set to four individuals for *D. cochinchinensis* and five individuals for *D. oliveri* (equivalent to the minimum sample size in the datasets). Arlequin was used to compute *F*_IS_ values for each population and locus, and values were tested for deviation from zero with 1000 permutations, using α = 0.05, and sequential Bonferroni procedure was conducted to calculate table‐wide levels of significance (Holm, 1979).

#### 2.3.2 Tests for genetic bottlenecks

To test for any evidence of genetic bottlenecks in the populations, we estimated the *T*_2_ statistic implemented in the Bottleneck program (Cornuet & Luikart, 1996), using the two‐phase mutation model (TPM) and default settings (var = 0.30, and proportion of stepwise mutation model (SMM) in TPM = 0.70), a one‐tailed Wilcoxon's signed rank test and 1000 iterations, and applying sequential Bonferroni corrections. The *T*_2_ statistic represents the deviation between observed and expected heterozygosity based on the number of observed alleles and is expected to be positive (heterozygote excess) if the population has experienced a recent bottleneck and negative (heterozygote deficiency) if the population has experienced a recent expansion. We also calculated the Garza–Williamson index *M*_m_ (Garza & Williamson, 2001) in Arlequin, calculated as $M_{m}=k/\left(r+1\right)$, where *k* is the number of alleles at a given locus, and *r* is the allelic range. Populations that went through bottlenecks are expected to have lost alleles, which is why the number of alleles is expected to be small in relation to the allelic range, yielding low *M*_m_ values. *M* is believed to better detect old and /or prolonged bottlenecks, while the *T*_2_ statistic detects more recent and less severe bottlenecks (Williamson‐Natesan, 2005).

#### 2.3.3 Genetic differentiation

To explore the level of genetic differentiation among populations, we used GenAlEx to calculate three global measures of differentiation: *G*_ST_ (adjusted for bias, following Meirmans and Hedrick (2011)), Jost′s *D* (Jost, 2008), and *G′′*_ST_ (Hedrick, 2005). All three values were tested with 999 permutations and 999 bootstraps. We also calculated pairwise *F*_ST_ values among all populations in Arlequin (Reynolds, Weir, & Cockerham, 1983).

To test the dataset for isolation by distance (IBD) among populations, a Mantel test (1967) as implemented in GenAlEx was conducted between a pairwise *F*_ST_ matrix and a matrix of pairwise geographical distances, and the significance tested with 9999 permutations.

STRUCTURE (Pritchard, Stephens, & Donnelly, 2000) was used to determine the most likely number of genetic clusters *K* for each species. We used the default settings (admixture model, the correlated allele frequencies model, and no prior population information). The likelihood for each *K* from 1–10 was estimated with 10 iterations per *K*, and a burn‐in of 20.000 MCMC and a simulation run of 200 000 MCMC for each iteration. In order to calculate the average likelihood value of each *K* over the 10 iterations and estimate Δ*K* (Evanno, Regnaut, & Goudet, 2005), the data were treated by the StructureHarvester web program (Earl & Vonholdt, 2012). The first analyses showed strong support for *K* = 2 for both *D. cochinchinensis* and *D. oliveri*. Further substructure was then investigated by performing a second round of analysis on the two clusters separately. For *D. oliveri*, there was a high level of admixture at both population and individual level between the two identified clusters. Therefore, admixture coefficients were first averaged over the 10 iterations for each individual with the software CLUMPP (Jakobsson & Rosenberg, 2007). The dataset was then divided according to *K* = 2 on an individual level, assigning individuals to a cluster if the admixture coefficient was 0.75 or above. For 46 individuals, no assignment was possible under these criteria, and they were omitted from the following analysis. Genetic structure within each of the two clusters identified for both species, respectively, was then explored with the same settings as in the main analysis.

For both species, CLUMPP was then used to average the outcome over the 10 iterations with the identified optimal number of clusters in the second round of analyses.

To analyze the partitioning of genetic variation at different hierarchical levels (within populations, among populations and among regions), we performed an AMOVA in Arlequin and tested the significance with 1000 permutations. For *D. cochinchinensis*, the five regions were defined by the clusters found in the second round of STRUCTURE analyses, which corresponded well to geographical location. For *D. oliveri*, where the clustering was not as well correlated to geography, the following six regions were chosen based on geography and the STRUCTURE results: 1. Laos (NIA, TXA, BAN, TKK), 2. north/central Cambodia (SMK, TBM, PL), 3. southwest Cambodia (RO, KRA, DP, KIR, DNT), 4. northeast Cambodia/ Central Highlands Vietnam (RBL, LUM, CMR, YOD), 5. southeast Cambodia/south Vietnam (SEI, CYS, LUS), and 6. southern part of Cat Tien National Park in Vietnam (DAL, CTA, CTB, SAM).

To include geographical location of populations as parameter, we conducted an additional genetic clustering analysis in Geneland (Guillot, Estoup, Mortier, & Cosson, 2005; Guillot, Mortier, & Estoup, 2005). We used population GPS coordinates for all samples and allowed an uncertainty of 2 km to the coordinates. Ten independent runs were conducted, with the correlated frequency option, null allele model, 200,000 iterations, a thinning of 100, and allowing the number of cluster *K* to vary between 1 and 12. The run with the highest posterior probability was chosen and treated with a burn‐in of 200.

#### 2.3.4 Mating system analysis

Genotypes of germinated seedlings and their mother trees were used to estimate outcrossing rates for the two populations of *D. cochinchinensis* and one population of *D. oliveri*. We used the software MLTR 2.2 (Ritland, 2002), which uses maximum‐likelihood computation under the mixed mating model, to calculate the following parameters: the multilocus outcrossing rate (*t*_m_), the average single‐locus outcrossing rate (*t*_s_), and the difference between these (at population level only) (*t*_m_ − *t*_s_), and *r*_p_, the correlation of paternity. If *t*_m_ is larger than *t*_s_, it is an indication of biparental inbreeding. We used the Newton–Raphson method and estimated standard errors of the calculated parameters with resampling of individuals within families based on 1000 bootstrap iterations.

## 3 RESULTS

### 3.1 Genetic diversity

We genotyped 677 individual samples of *D. cochinchinensis* and 616 samples of *D. oliveri*, but only found 523 and 465 unique genotypes, respectively.

The Probability of Identity (PI) showed that identical genotypes were likely to represent clones of the same individual (PI values from 1.5E^−3^ to 5.7E^−10^ for *D. cochinchinensis* and 1.6E^−4^ to 7.7E^−10^ for *D. oliveri*). Levels of clonality differed greatly among populations, with several populations having clonal richness estimates of 1 or close to 1 (1 =  all individuals had different genotypes) and the ANG *D. cochinchinensis* population as low as 0.16. For both species, especially populations from southwest Cambodia had very low clonal richness (DP, RO, KRA, DNT) (Table 1). However, as sampling strategy and geometry can influence these estimates and actual spatial distribution of sampled individuals varied among sites depending on density of accessible individuals, comparison among populations should be interpreted with caution.

No linkage disequilibrium was found among the developed microsatellite markers except for a single marker combination in the *D. oliveri* SEI population, and we found no evidence of scoring errors in the dataset. Tests for HW proportions showed significant deviations at three loci/population combinations each for *D. cochinchinensis* and *D. oliveri,* and both species also showed some evidence for null alleles.

Comparison of *F*_ST_ values of the original data and the data corrected for null alleles showed that any bias was minor, and we therefore conducted all further analyses on the original datasets (original/corrected *F*_ST_ values*: D. cochinchinensis*: 0.247 / 0.236 and *D. oliveri:* 0.128 / 0.126).

Overall levels of expected heterozygosity indicated medium levels of genetic diversity for *D. cochinchinensis* (mean *H*_e_ = 0.55) and high levels for *D. oliveri* populations (mean *H*_e_ = 0.73) (Table 1). In both species, populations in the center of the distribution area had the highest level of diversity, with decreasing levels toward the peripheral populations. This pattern was most pronounced for *D. cochinchinensis* with levels of diversity differing greatly among regions. The highest levels of diversity for this species was observed around the Dangrek mountains in the northern border area between Cambodia and Thailand (populations PJ to NOY with *H*_e_ around 0.70), followed by the Cardamom and Elephant mountains area (populations RO to SPEU with *H*_e_ from 0.61–0.66). The Vietnamese and the three northeastern Thai populations had intermediate diversity levels (*H*_e_ = 0.47–0.50), and the lowest values were found for the Laotian populations (*H*_e_ = 0.34–0.43).

The Cambodian populations of *D. oliveri* in general had the highest *H*_e_ values, almost all around 0.8, which was also found for the two northernmost Vietnamese populations, CMR and YOD. The Laotian populations had slightly lower *H*_e_ from 0.68–0.79, while the Cambodian SEI and southern Vietnamese populations had considerably lower levels of diversity (*H*_e_ = 0.58–0.67). For both species, allelic richness measured both by raw number of alleles and adjusted by sample size generally showed the same regional patterns.

We did not detect any signs of inbreeding in either species, as *F*_IS_ averaged at 0.004 for *D. cochinchinensis* and at −0.017 for *D. oliveri*, with no population values differing significantly from zero. For *D. cochinchinensis*, the positive value in population PK could be due to the effect of null alleles present in this population, as is also the case for the PW, TAL, and NIA populations (Table 1).

The *M*_m_ values for both species were all low (0.23–0.48), well below the critical values of 0.68 (Garza & Williamson, 2001), indicating that all populations went through old or prolonged bottlenecks. *T*_2_ values varied among populations and only one *D. cochinchinensis* (ANG) and three *D. oliveri* populations (TXA, RBL, TBM) showed signs of recent bottlenecks; although none were significant after adjusting with table‐wide sequential Bonferroni.

### 3.2 Genetic differentiation among populations

We found a high level of differentiation among populations of *D. oliveri* (Global *G*_ST_ = 0.13) and a very high level among populations of *D. cochinchinensis* (Global *G*_ST_  = 0.25). The alternative estimators of genetic differentiation, *G′′*_ST_ and Jost′ *D*, which are not affected by level of heterozygosity (Meirmans & Hedrick, 2011), however indicated almost equally high levels of differentiation for the two species (Table 2) (all values highly significant).

**Table 2:** Genetic differentiation measures for *Dalbergia cochinchinensis* (26 populations) and *Dalbergia oliveri* (23 populations). The AMOVA additionally included five regions for *D. cochinchinensis* and six for *D. oliveri* (see Materials and Methods for details on regions)

	*D. cochinchinensis*	*D. oliveri*
*G*_ST_	0.25	0.13
*G′′*_ST_	0.59	0.55
Jost′ *D*	0.45	0.49
AMOVA *F*_ST_	0.27	0.13

The AMOVA showed particularly a stronger regional differentiation for *D. cochinchinensis* than for *D. oliveri*, as *D. cochinchinensis* had 17% of genetic variation located among regions and 10% among populations and within regions, while for *D. oliveri,* 7% of genetic variation was located among regions and 7% among populations and within regions (Tables S1, S5 and S6).

**Figure 2:**
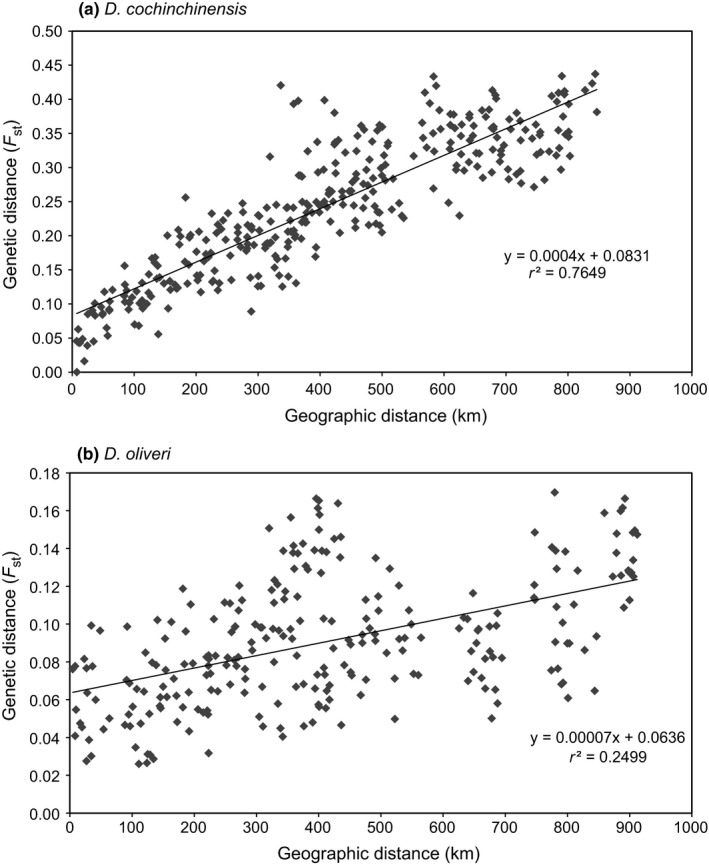
Isolation by distance among populations of (a) *Dalbergia cochinchinensis* and b) *Dalbergia oliveri*. Regression equations shown in graphs, *p *= .0001 for both species

Pairwise population *F*_ST_ values were between 0–0.45 for *D. cochinchinensis* and 0–0.25 for *D. oliveri* and were highest among geographically distant populations (Figs S2 and S3). The IBD analysis confirmed a highly significant relationship between *F*_ST_ values and geographical distance between populations for both species, although much stronger for *D. cochinchinensis* (*r* ^2^  = .76, *p *= .0001) than for *D. oliveri* (*r* ^2 ^= 0.25, *p *= .0001) (Figure 2). An exception from this pattern was a strong differentiation between southern Vietnamese populations of *D. oliveri* (CYS to SAM) and the populations in southwest Cambodia (RO to DNT) (Fig. S3).

When using STRUCTURE to analyze the partitioning of genetic variation the first analyses showed that *K *=* *2 was the most likely number of genetic clusters overall, for both species. For *D. cochinchinensis,* these two clusters divided the populations into a north (All Lao and northernmost Thai populations) and a south group (Cambodian, Vietnamese, and the PJ Thai population) with very limited admixture between them (Fig. S4). For *D. oliveri, K *=* *2 roughly separated the southeastern populations (SEI, YOD, CYS, LUS, DAL, CTA, CTB, SAM) from the rest, but with a high level of admixture on both population and individual level (Fig. S5).

**Figure 3:**
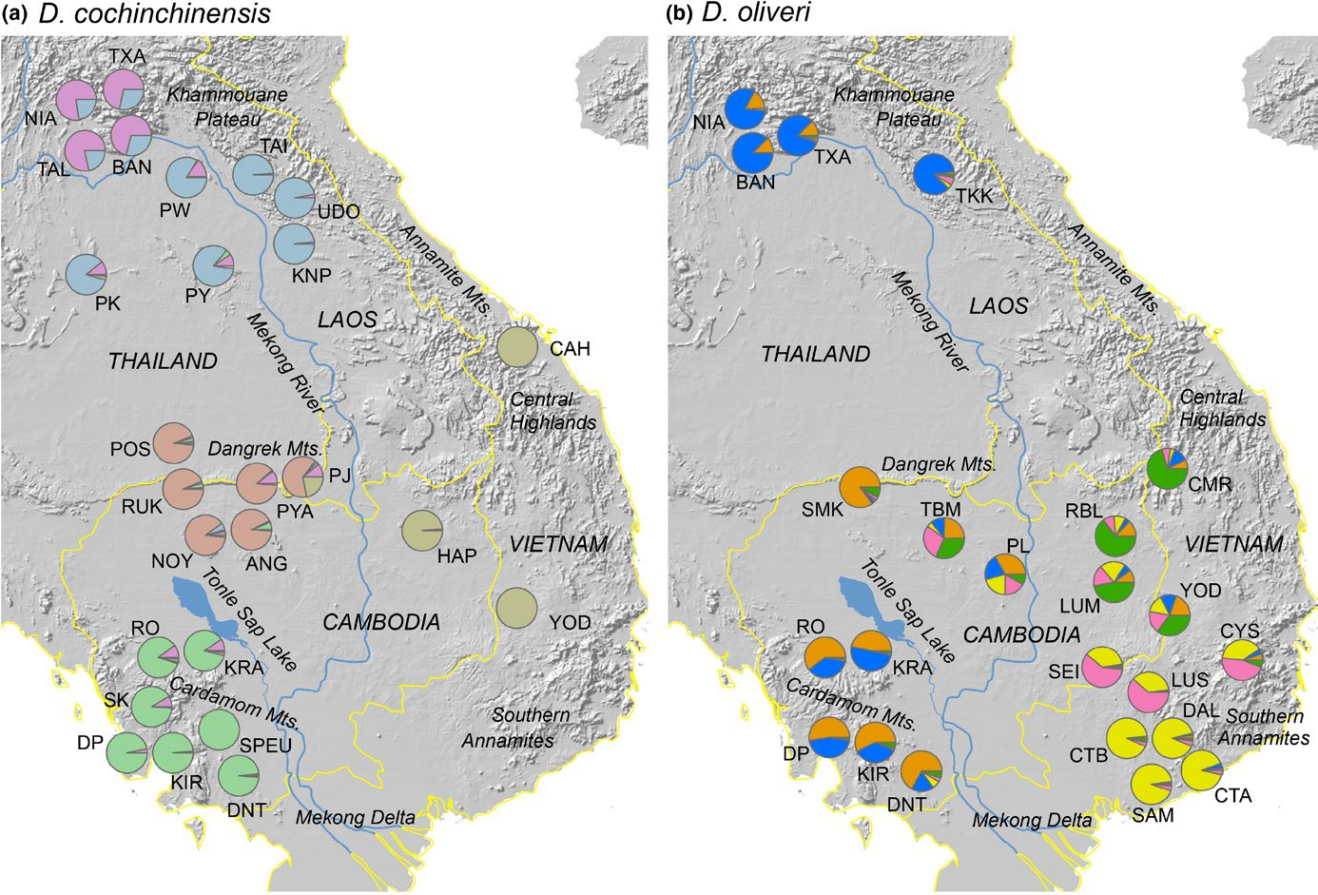
Structure results for *K *=* *5 for (a) *Dalbergia cochinchinensis* and (b) *Dalbergia oliveri*. The allocation of individuals within each population to the five identified clusters for each species is shown in different colors

**Figure 4:**
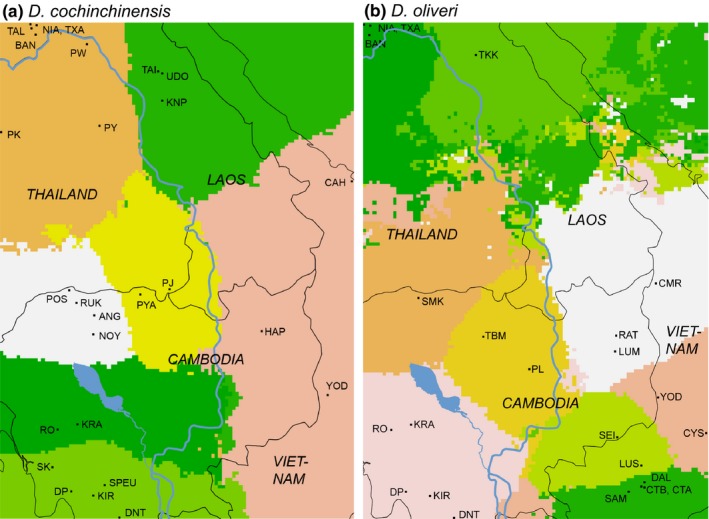
Geneland results for (a) *Dalbergia cochinchinensis* and (b) *Dalbergia oliveri*. Geneland outputs overlaid with map of country borders and major water bodies in the region

Further analysis of substructure revealed a total of five clusters each for both species (Figure 3). The Geneland analyses suggested a higher number of clusters: seven for *D. cochinchinensis* and nine for *D. oliveri* (Figure 4).

For *D. cochinchinensis*, the STRUCTURE analysis showed a strong geographical correlation with genetic clusters as the majority of populations had more than 80% of the individuals assigned to a single cluster (Figure 3). The 26 populations can thus be divided into the corresponding five clusters: 1. Northern Laotian populations (NIA, TXA, TAL, BAN), 2. Central Laotian and the northernmost Thai populations (TAI, UDO, KPN, PW, PK, PY), 3. Dangrek mountain region (POS, RUK, ANG, NOY, PYA, PJ), 4. southwest Cambodian populations (RO to DNT), and 5. east Cambodian/Vietnamese populations (HAP, CAH, YOD). The Geneland analysis generally agreed with this pattern, but further divided the southwest Cambodian populations into northern Cardamoms (RO, KRA) and southern Cardamoms/Elephant Mountains (SK to DNT), and the Dangrek mountain area group into a western (POS to NOY) and eastern group (PYA, PJ). In the northern part of the sampling area, the Geneland analysis rather separated the Central Laotian from the Northern Thai and Laotian populations (Figure 4).

For *D. oliveri*, the STRUCTURE analysis yielded a more admixed structure than for *D. cochinchinensis*. Only two groups at the periphery of the sampling area, the Laos populations (NIA to TKK) and the most SE Vietnamese populations (DAL to SAM) were almost exclusively assigned to a single cluster, while the remaining populations were more admixed. However, the SEI, CYS, and LUS populations can be recognized as a group from the same patterns of admixture, as can the SW Cambodian populations (RO to DNT) and the eastern Cambodian and central Vietnamese populations (RBL, LUM, CMR, YOD). In the center of the sampling area, the Cambodian populations TBM and PL shared some patterns, while the SMK population appeared quite distinct (Figure 3).

The Geneland analysis overall found the same groups as the STRUCTURE analysis and particularly confirmed the distinctiveness of the SE Vietnamese populations from the southern part of Cat Tien National Park in relation to the geographically adjacent populations LUS (located in the northern part of Cat Tien National Park), SEI and CYS, as well as of the SMK population. However, it differed in the separation of TKK from the other Laotian populations, and the clustering of the Vietnamese YOD + CYS. The unresolved patterns in the area corresponding to Thailand, southern Laos, and Central Vietnam are probably due to the large geographical gap in sampling between the Laotian populations and the rest (Figure 4).

For both *D. cochinchinensis* and *D. oliveri*, the Geneland maps present suture zones running north–south roughly on the location of the Mekong River, thus proposing this as an important barrier to gene flow in both species. Suture zones are also found at the approximate location of the Tonle Sap Lake and River, isolating the southwestern Cambodian populations (RO‐DNT) from the rest, although most pronounced for *D. oliveri* (Figure 4).

### 3.3 Mating system

The mother genotypes revealed that some of the *D. cochinchinensis* trees were clones (cf results above), and the offspring from these trees were therefore pooled, resulting in 16 families for the NOY population and 11 families from the SPEU population.

Average germination success for *D. cochinchinensis* was 22% for the NOY population and 36% for the SPEU population and 37% for the *D. oliveri* RBL population, with highly variable individual germination rates for both species.

Whereas the *D. oliveri* seed genotypes showed a realized estimated outcrossing rate of 95% indicating almost exclusive outcrossing, some levels of selfing was found in both *D. cochinchinensis* populations with outcrossing rates of 82% and 71%, respectively (Table 3).

**Table 3:** Estimation of outcrossing rate for two populations of *Dalbergia cochinchinensis* (NOY and SPEU) and one population of *Dalbergia oliveri* (RBL). Average over individuals, SD in parentheses

	*t*_m_	*t*_m_ *−t*_s_	*r*_p_multilocus	1/*r*_p_
*D. cochinchinensis* NOY	0.824 (0.050)	0.101(0.029)	0.154 (0.044)	6.5
*D. cochinchinensis* SPEU	0.710 (0.042)	−0.011(0.025)	0.049 (0.045)	20.4
*D. oliveri* RBL	0.951 (0.020)	0.074 (0.035)	0.143 (0.044)	6.99

The *D. cochinchinensis* NOY populations and the *D. oliveri* RBL population both showed indication of biparental inbreeding, and a low number of effective fathers, while the *D. cochinchinensis* SPEU population had a higher number of effective fathers and no indication of biparental inbreeding (Table 3).

## 4 DISCUSSION

Indochina represents a biodiversity hot spot but at the same time the area is severely under‐described and the knowledge of intraspecific variation within its endemic species very limited. To our best knowledge, the present study represents the first assessment of region‐scale patterns of genetic structure and diversity of populations of a tree species across central Indochina. Several interesting trends common for both *Dalbergia* species emerged from the data. Both species showed a pattern of higher levels of genetic diversity in the central parts of the sampling area and a distinct regional genetic differentiation. The patterns of genetic clustering across Indochina were largely shared between the two species and thus reflected impacts of landscape features such as the Mekong River and the Tonle Sap Lake. On the other hand, we also found substantial differences between the two species. *Dalbergia oliveri* had a higher level of genetic diversity and a lower level of geographical differentiation than *D. cochinchinensis*, which could reflect differences in life‐history traits.

Specifically for *Dalbergia*, our data further revealed that both species reproduced clonally. We assumed that root suckers were the primary mode of clonal reproduction in these species, as such were directly observed at several localities, for both species. This ability of clonal reproduction has been proposed earlier for *Dalbergia* (So, Theilade, & Dell, 2010) and also matches well with observations that the species shows strong coppice regeneration from stumps after logging (CoP16 C, 2013; CTSP 2004). Further exploration on the aspect of clonality is considered beyond the scope of this paper, and below we will focus on the common trends in population genetic diversity and structure and discuss how these can be related to geography and history of the Indochinese landscape, as well as to recent deforestation levels. We also discuss how the differences between the two species might relate to differences in life‐history traits.

### 4.1 Population genetic structure of *D. cochinchinensis* and *D. oliveri* —can be explained by the Indochinese landscape

The population genetic structure of both species as reflected by STRUCTURE and GENELAND analyses showed patterns that corresponded well to landscape features of the Indochina region. The greatest dispersal barrier seemed to be the southern part of the Mekong River as well as the Mekong Delta, as these effectively separated populations on either side in different genetic clusters (Figures 3 and 4). For *D. oliveri,* these water bodies seemed to represent such a strong dispersal barrier, that it left the populations in the Cardamom region in southwest Cambodia more genetically related to the distant populations in Laos than to the much more adjacent populations in the Southern Annamite region of Vietnam (Figures 3 and S3). In fact, the populations in the south easternmost Vietnam (from southern part of Cat Tien National Park) were surprisingly different from the populations just to the north of them, indicating a long history of isolation and limited gene flow. This region is bordered by sea to the south and east, and the Mekong River Delta to the west, which means that populations are only connected to the rest through the north. This could explain the very low levels of genetic diversity found in *D. oliveri* populations in this region. Interestingly, a study of an Indomalayan tree mouse showed parallel results of a highly diverged and low diversity South Vietnamese lineage (Meschersky, Abramov, Lebedev, Chichkina, & Rozhnov, 2016), similarly located to the forest area around and in the Cat Tien National Park. The authors also interpreted this distinctiveness as a result of the locality being a “closed pocket” between the sea and the river, with a related high degree of isolation.

The lower Mekong and the Mekong Delta have been recognized as a biogeographical barrier to distribution of mammal and amphibian species with a high diversity of endemics found east of the Mekong (Geissler et al., 2015; Meijaard & Groves, 2006). The present course of the Mekong River is only around 5000 years old (Woodruff, 2010); however, the proximate location of the Lower Mekong has been separating the Annamite Mountains from the Cardamom Mountains since late Miocene (Geissler et al., 2015; Hutchison, 1989).

The Mekong River acting as both an ancient and current landscape barrier to gene flow can explain the high divergence level between Cardamom and Annamite populations of *D. oliveri*. The results in this study thus confirm the presence of the lower Mekong as an important barrier to dispersal, also at intraspecific level.

In addition, the great Tonle Sap Lake in Cambodia also appears to act as a dispersal barrier for both species, with suture zones at its proximate location and considerable differences in genetic cluster allocation on either side of the lake (Figures 3 and 4). Tonle Sap is surrounded by large seasonally inundated zones, and the combination of the two thus represents a large area without suitable habitat for either *Dalbergia* species.

In the northern part of the sampling range, the barrier effect of the Mekong River is not as clear. This suggests that it is not the river itself that represents a dispersal barrier to the two *Dalbergia* species but rather the addition of water bodies and adjacent wetland habitats that limits effective gene flow.

Similar to our findings, the Red River, that runs from Yunnan province in China through northern Vietnam and exits into the Gulf of Tonkin, has also been found to act as dispersal barrier to a plant species, creating intraspecific differentiation (Zhao & Zhang, 2015).

### 4.2 Ancient imprints on current genetic diversity levels in *D. cochinchinensis* and *D. oliveri*

Common for both species was an apparent reduced genetic diversity in more peripheral populations compared to the most diverse populations in Cambodia, although most pronounced for *D. cochinchinensis*. Hansen et al. (2015) also found large regional differences of diversity in teak (*Tectona grandis)* across the distribution range from India to Laos, with decreasing diversity along a west–east gradient, and interpreted the pattern as an indication that teak has its center of origin in India and then spread eastwards. The patterns for *D. oliveri* and *D. cochinchinensis* may also be ancient, reflecting the area of origin of the two species or perhaps reflect common refugia during earlier time periods with reduced forest cover, from where the species spread to more peripheral regions. In both cases, repeated founder effects during initial colonization or recolonization from refugia would be responsible for the reduced diversity toward the edges of the distribution area.

The low values of *M*_*m*_ for both species could indicate that they experienced reduced population sizes due to forest fragmentation during the earlier time periods, *that is,* the Pleistocene. During the Pleistocene, glacial periods caused dry and cool conditions in mainland SE Asia, which according to several studies resulted in substantial (rain) forest contraction and fragmentation, accompanied by extension of grassland and savannah habitats (Bird et al., 2005; Heaney, 1991; Hope et al., 2004). Other studies, though, indicate that larger areas of lowland forest still persisted during glacial maxima, partly due to the larger land areas exposed by lower sea levels, and that the current extension of tropical rainforest actually is the refugial stage (Cannon, Morley, & Bush, 2009; Wang, Sun, Wang, & Stattegger, 2009). Evidence for either theory within the area covered in this study seems limited by number and distribution of analyzed sediment cores that can be dated back to glacial periods (see eg. Kershaw, Penny, Van der Kaars, Anshari, & Thamotherampillai, 2001; Maxwell & Liu, 2002). Only a few studies have analyzed the demographic history of forest species covering the same area as in the present study and linked it to hypotheses of earlier forest distribution patterns. Morgan et al. (2011) found that nine forest‐associated mosquito species from mainland SE Asia showed strong evidence for refugia in northern mountainous areas of the region during Pleistocene glacial periods, and Fuchs et al. (2008) suggested that isolation in mountainous refugia in earlier cooler periods caused the presence of several distinct lineages within a forest bird in Indochina. However, Latinne et al. (2015) found that forest‐dwelling rodents species of SE Asia show evidence of range contraction during interglacial rather than glacial periods and suggested that the southern part of Indochina may have acted as refugium, as well as northern parts.

We found no clear signs of northerly located refugia for the two *Dalbergia* species, as these areas generally harbored lower levels of genetic diversity. The two clearly separated north and south clusters found for *D. cochinchinensis* in the first level of STRUCTURE analysis could though be interpreted as the result of an earlier separation into northern (e.g., Laos/Northeastern Thailand) and southern (e.g., Central Cambodia/Vietnam) refugia. The populations in the northern cluster showed slightly higher *M*_m_ values than the southern populations, which could indicate a less severe bottleneck, maybe due to larger and more continuous forest refugia supported by the vast highland areas of northern Laos and Vietnam. However, the existing north/south pattern is also likely a result of the high degree of IBD in *D. cochinchinensis* and the deficiency of sampling in the area between the clusters. In general, there was a tendency that the highest levels of diversity were found in the mountainous regions and surrounding lowland areas of the Cardamom mountains (Cambodia), the Dangrek mountains (Thai/Cambodian border) and the Central Annamites/Highlands (Vietnam). It could indicate that these areas have had a continuous presence of suitable forest habitats for *Dalbergia*, persisting through earlier time periods such as glacial cycles. As the preferred habitat for *D. cochinchinensis* is deciduous and semi‐deciduous forest, and semi‐deciduous to evergreen forests for *D. oliveri,* it could explain why there is less overlap with refugia found for rainforest‐associated species. According to Cannon et al. (2009), the area corresponding to the Gulf of Thailand were exposed during the last glacial maximum and was covered by semi‐deciduous forests. The distribution of both *D. cochinchinensis* and *D. oliveri* could thus have extended southwards of the present distribution during glacial periods, which makes the current distribution the more restricted one. This hypothesis seems more likely for *D. oliveri*, with matching high levels of diversity found in the Cardamom mountains area.

### 4.3 Effect of deforestation and fragmentation

Indochina has been experiencing high levels of deforestation approximately since the middle of the 20th century, with current levels among the world′s highest (FAO 2009; Lestrelin, 2010; Meyfroidt & Lambin, 2008; SCW 2006; WRI 2015), and it could thus be anticipated that forest trees as *Dalbergia* would suffer from reduced genetic diversity and higher levels of inbreeding (Lowe et al., 2004). Studies of other tropical timber *Dalbergia* species have indeed found lower levels of heterozygosity in populations from more fragmented areas opposed those found in less fragmented forests (Andrianoelina et al., 2006; Resende, Ribeiro, & Lovato, 2011). However, due to long and overlapping generation times and generally high levels of outcrossing and dispersal rates, trees might not respond immediately to fragmentation in terms of loss of genetic diversity (Davies et al., 2015; Kramer, Ison, Ashley, & Howe, 2008; Lowe, Boshier, Ward, Bacles, & Navarro, 2005). We found moderate to high levels of genetic diversity in *D. cochinchinensis* and *D. oliveri,* comparable to those of other trees species from the area (Pakkad et al., 2014; Senakun et al., 2011; Wattanakulpakin et al., 2015), and no evidence of inbreeding in any populations (no *F*_IS_ values significantly different from zero, Table 1). Further, the *T*_2_ statistic, which detects loss of alleles not yet matched by a loss of heterozygosity, did not reveal any significant signs of a recent bottleneck. Therefore we believe that forest fragmentation and targeted logging have not yet had a crucial impact on the observed genetic diversity of the two species.

While effects on genetic diversity levels might be delayed to later generations, more immediate consequences of fragmentation have been identified in the mating system of neotropical trees (Lowe et al., 2005). Several studies have shown evidence of decreases in pollen diversity and increases in selfing rates for insect‐pollinated trees, with increased levels of fragmentation and logging (Breed et al., 2012, 2015; Obayashi et al., 2002). In 1994, Sonhuua et al. estimated outcrossing rates of 0.99 for eight different Thai populations of *D. cochinchinensis*. In the present study, we also found that both *Dalbergia* species were primarily outcrossing; however, *D. cochinchinensis* showed selfing rates of around 20–30% in the two studied populations (NOY and SPEU). There were no signs of inbreeding (high *F*_IS_) in the adult generation from these two populations perhaps suggesting strong selection against selfed progeny due to inbreeding depression in the natural populations. It is, however, also possible that the observed significant levels of selfing represented a very recent change in the species’ mating system as an immediate response to severely reduced population size. Outcrossing rates can differ both spatially and temporary (Eckert et al., 2010; Goodwillie, Kalisz, & Eckert, 2005), and as our estimates are based on few samples in a single year, the interpretation should be cautious.

### 4.4 Differences between *D. cochinchinensis* and *D. oliveri* can be explained by differing life‐history traits

*Dalbergia oliveri* seems to have a higher level of gene flow than *D. cochinchinensis,* which was shown by a higher and more evenly distributed level of genetic diversity and a more admixed population structure. Both species showed a markedly higher level of genetic differentiation than other Indochinese trees (Pakkad, Ueno, & Yoshimaru, 2008; Pakkad et al., 2014; Senakun et al., 2011; Wattanakulpakin et al., 2015) or *Dalbergia* species (Andrianoelina, Favreau, Ramamonjisoa, & Bouvet, 2009; Resende et al., 2011). The levels found for *D. cochinchinensis* corresponded relatively well with earlier estimations of differentiation for the species (Moritsuka et al., 2013; Soonhua et al., 1994).

The two species are assumed to be pollinated by small insects, which presumably have limited pollen dispersal capability at longer distances, and thus may only be of significance within populations. Gene flow through dispersal of pollen is not expected to differ much between the two species. With regard to seed dispersal, both species are thought to be spread by wind and/or possibly water, although water dispersal seems more likely for *D. oliveri* than for *D. cochinchinensis*. The fruits of *D. oliveri* are larger and more hard/leathery than those of *D. cochinchinensis*, which are thinner and more papery. Further, the seed spaces are greater and hollower in pods of *D. oliveri* and have thicker walls. These differences in pod morphology could make *D. oliveri* fruits better at floating along streams and rivers, promoting dispersal. *Dalbergia oliveri* trees are often found along streams and rivers, a fact that seems to support frequent water dispersal of *D. oliveri*. A genuine difference in dispersal strategy and ability between the two species could help explain the higher degree of isolation by distance, lower level of population admixture as well as the lower levels of genetic diversity in *D. cochinchinensis* compared to *D. oliveri*.

High levels of gene flow may counteract the negative consequences of fragmentation and logging, thus mitigating the loss of genetic diversity (Bacles, Lowe, & Ennos, 2006; Davies et al., 2015; White, Boshier, & Powell, 2002). If the actual level of inter population gene flow is indeed higher for *D. oliveri* than for *D. cochinchinensis*, the effects of deforestation would differ among the two, making *D. oliveri* relatively more resilient and *D. cochinchinensis* more vulnerable to logging and fragmentation in the long term.

*Dalbergia cochinchinensis* is described as intermediate pioneer species (So, 2000), with higher growth rates than *D. oliveri* at young stages (So Thea, pers. obs.). Pioneer characteristics include ability of rapid colonization of new habitats only with a few individuals, and higher frequency of such founder events may explain the lower diversity in populations of *D. cochinchinensis* compared to *D. oliveri* and would also result in higher differentiation levels among populations.

As selfing species generally have lower levels of genetic diversity and higher levels of differentiation than outcrossing species (Hamrick & Godt, 1996), the 20–30% selfing rate estimated for *D. cochinchinensis* could also be a factor responsible for different differentiation and diversity levels between the two species. However, as there were no signs of inbreeding in the adult populations of *D. cochinchinensis (see* *discussion* *above)*, we do not consider it likely that selfing caused the observed differences among the two species.

Finally, *D. oliveri* has a broader distribution area and occurs in a wider ecological niche than *D. cochinchinensis* (CTSP 2004; Niyomdham, 2002; Niyomdham et al., 1997), thus supporting a larger total population size than *D. cochinchinensis* (assuming approximate similar densities). This could also explain the higher diversity levels in *D. oliveri* (Hamrick & Godt, 1996).

Despite the apparent differences in life‐history traits and levels of genetic diversity and geographical differentiation between the two *Dalbergia* species, both species exhibit common characteristics in population structure, which can be explained by current and ancient landscape patterns in Indochina. The present study provides an initial assessment of intraspecific genetic diversity patterns in Indochina, and it is likely that the landscape effects of, for example, the Mekong River and Tonle Sap Lake found for the *Dalbergia* species will also be reflected in other Indochinese species. Further studies with other focal species will reveal more about current and ancient landscape effects on the distribution and diversity of species in this highly diverse but also highly threatened region.

## CONFLICT OF INTEREST

None declared.

## AUTHOR CONTRIBUTIONS

IH, ST, SC, HTT, SB, IT, EDK, and LRN involved in study concept and design. IH, SC, and HTT involved in fieldwork and sample acquisition. IH, SC and HTT involved in laboratory work. IH, EDK, and LRN involved in analysis and interpretation of data. IH and LRN involved in drafting of manuscript. IH, ST, SC, HTT, SB, IT, EDK, and LRN involved in critical revision.

## DATA ACCESSIBILITY

Primer and microsatellite sequences are deposited in GenBank accession number KR558727‐KR558743. Microsatellite genotypes can be found at Dryad Digital Repository: doi.org/10.5061/dryad.2dg7c.
