# Supplementary material for: Population genetic structure of the endemic rosewoods Dalbergia cochinchinensis and D. oliveri at a regional scale reflects the Indochinese landscape and life‐history traits
Source: Ecol Evol. 2017 Dec 1;8(1):530–45. doi: 10.1002/ece3.3626 (PMC5756888; doi:10.1002/ece3.3626)
Supplement: Supplementary file 1 [file ECE3-8-530-s001.pdf]

Supporting information for:

Population genetic structure of the endemic rosewoods *Dalbergia cochinchinensis* and *D. oliveri* at a regional scale corresponds to the Indochinese landscape and life history traits

Ida Hartvig[1*], Thea So[2], Suchitra Changtragoon[3], Hoa Thi Tranh[4], Somsanith Bouamanivong[5], Ida Theilade[6], Erik Dahl Kjær[1], Lene Rostgaard Nielsen[1]

[1]Department of Geosciences and Natural Resource Management, University of Copenhagen, Rolighedsvej 23, 1958 Frederiksberg C, Denmark

[2] Institute of Forest and Wildlife Research and Development, Forestry Administration, Ministry of Agriculture Forestry and Fisheries, No. 1017, Hanoi Street, Sen Sok District, Phnom Penh, Cambodia 12101

[3]Forest and Plant Conservation Research Office, Department of National Parks, Wildlife and Plant Conservation, Ministry of Natural Resources and Environment, 61 Phaholyothin, Chatuchak, Bangkok, Thailand 10900

[4]Forest Genetics and Conservation, Center for Biodiversity and Biosafety, Institute of Agricultural Genetics, Vietnam Academy of Agricultural Sciences, Pham Van Dong Road, Hanoi, Vietnam

[5]National Herbarium of Laos, Biotechnology and Ecology Institute, Ministry of Science and Technology, Ventiane, Laos

[6]Department of Food and Resource Economics, University of Copenhagen, Rolighedsvej 21, 1958 Frederiksberg C, Denmark

* Corresponding author: ihla@ign.ku.dk

Table S1 Sample and locality information for the 26 *D. cochinchinensis* populations included in this study. NP = National Park

Country	Pop	Province	Locality	Latitude N	Longitude E	Elevation (m a.s.l.)	DBH (cm)	# Samples
**Laos**	**NIA**	Bolikhamsai	Phu Khao Khuay NP, Da Niai	18.46043	103.05003	507	5.2	25
	**TXA**	Bolikhamsai	Phu Khao Khuay NP, Tad Xai	18.45269	103.14494	331	13.2	16
	**TAL**	Bolikhamsai	Phu Khao Khuay NP, Tad Leuk	18.39756	103.07171	205	9.6	22
	**BAN**	Bolikhamsai	Phu Khao Khuay NP, Ba Na	18.30135	103.13253	182	7.4	28
	**TAI**	Khammouane	Na Kai Tai	17.74779	105.09066	633	8.8	25
	**UDO**	Khammouane	Udomsuk	17.72729	105.16033	541	10.7	28
	**KPN**	Khammouane	Khaun Pheung Neua	17.30668	105.15304	276	11.1	26
**Thailand**	**PW**	Bueng Kan	Phu Wua Wildlife Sanctuary	18.16001	103.95249	230	-	30
	**PK**	Nongbualamphu	Phukao-Phuphankham NP	16.81065	102.61121	200	-	29
	**PY**	Sakon Nakhon	Phu Pha Yon NP	16.92574	104.16688	230	-	33
	**PJ**	Ubon Ratchathani	Phu chong-nayoi NP	14.43842	105.25815	240	-	22
**Cambodia**	**POS**	Otdor Meanchey	Phnom O´Smach	14.41977	103.69363	249	11.4	30
	**RUK**	Otdor Meanchey	Rukkavan	14.2317	103.81054	50	9.7	33
	**ANG**	Otdor Meanchey	Anglong Veng	14.04555	104.09153	84	8.4	20
	**NOY**	Siem Reap	Sre Noy	13.75205	104.08103	84	30.8	27
	**PYA**	Preah Vihear	Prey Eth	14.36245	104.81184	125	6.8	32
	**RO**	Pursat	Rovieng	12.31026	103.52344	133	8.1	25
	**KRA**	Pursat	Kravanh	12.39044	103.83185	84	11.8	29
	**SK**	Koh Kong	Spin Kdah	11.72949	103.4464	418	5.1	30
	**DP**	Koh Kong	Daeng Peng	11.36869	103.7515	59	5.4	28
	**KIR**	Kampong Speu	Kirirom NP	11.30544	104.08611	480	2.7	29
	**SPEU**	Kampong Speu	Kampong Speu	11.46304	104.25334	63	19.8	28
	**DNT**	Kampot	Damnak Neakta Thmapoun	10.97373	104.48833	72	5.1	10
	**HAP**	Ratanakkiri	Hark Port	13.80253	106.69694	111	9.4	25
**Vietnam**	**CAH**	Da Nang	Cam Ha	16.05859	108.0322	-	-	6
	**YOD**	Dak Lak	Yok NP	12.82472	107.7178	203	-	41

Table S2 Sample and locality information for the 23 *D. oliveri* populations included in this study. NP = National Park

**Country**	**Pop**	**Province**	**Locality**	**Latitude N**	**Longitude E**	**Elevation (m a.s.l.)**	**DBH (cm)**	**# Samples**
**Laos**	**NIA**	Bolikhamsai	Phu Khao Khuay NP, Da Niai	18.46053	103.0496	497	14.9	11
	**TXA**	Bolikhamsai	Phu Khao Khuay NP, Tad Xai	18.4525	103.14477	320	32	17
	**BAN**	Bolikhamsai	Phu Khao Khuay NP, Ba Na	18.30295	103.13391	158	32.5	5
	**TKK**	Khammouane	Tha Khan Keo	18.00416	104.82488	253	29.8	24
**Cambodia**	**SMK**	Otdor Meachey	Samaki	14.31581	103.93433	88	10.1	33
	**TBM**	Preah Vihear	Tbeng Meanchey	13.72006	104.94944	146	17	39
	**PL**	Stung Treng	Prey Long	13.23103	105.66763	150	-	9
	**RO**	Pursat	Rovieng	12.31106	103.52557	83	2.5	29
	**KRA**	Pursat	Kravanh	12.38923	103.83306	114	14.4	28
	**DP**	Koh Kong	Daeng Peng	11.36781	103.75345	69	3.3	30
	**KIR**	Kampong Speu	Kirirom NP	11.31245	104.12987	114	3.3	30
	**DNT**	Kampot	Damnak Neakta Thmapoun	10.97467	104.48833	82	3.4	23
	**RBL**	Rattanakiri	Boeng Yeak Laom, Ban Lung	13.73426	107.0162	329	18.6	47
	**LUM**	Rattanakiri	Lumphat	13.49354	106.99189	85	15.7	33
	**SEI**	Mondulkiri	Keo Seima Protection Forest	12.19227	107.01539	317	22.3	36
**Vietnam**	**CMR**	Kon Tum	Chu Mom Ray NP	14.52286	107.65021	364	-	33
	**YOD**	Dak Lak	Yok Don NP	12.79678	107.65757	255	-	23
	**CYS**	Dak Lak	Chu Yan Sinh NP	12.24193	108.39529	644	-	56
	**LUS**	Lam Dong	Cat Tien NP, northern part	11.75582	107.39253	330	15.4	23
	**DAL**	Dong Nai	Cat Tien NP	11.50746	107.43525	180	20.5	24
	**CTA**	Dong Nai	Cat Tien NP, near park office	11.42992	107.4277	138	20.1	25
	**CTB**	Dong Nai	Cat Tien NP, Crocodile Lake	11.44548	107.3871	192	12.1	13
	**SAM**	Dong Nai	Cat Tien NP	11.36397	107.19138	101	6.6	25

Table S3 Characteristics of the nine microsatellite markers for *D. cochinchinensis* developed for this study. Product size range and number of alleles based on 677 samples.

Primer name	Repeat motif	Product size range	#Alleles	Primer sequences (5´-3´)	Fluorescent label	Genbank accession
COC_01	gt	177-200	11	F: TAATGGAAAACAGTCGAAACAA<br>R: TCCAGCTGAGATTCAAGCCT	VIC	KR558727
COC_05	ag	117-144	12	F: CCAACATTCACATCATTATCAGC<br>R: CCCCAAACTAGGTTTTTCTCA	VIC	KR558728
COC_06	ag	214-250	17	F: CCTACGATGTTCTACGGGGA<br>R: ACCGATGACTGATGAGGTGA	6-FAM	KR558729
COC_07	ag	134-166	16	F: AATAATCTTCCATTCCTTCTTTGC<br>R: TGAAGGACCAAGAGACACGG	PET	KR558730
COC_08	ac	133-170	17	F:TGATGATATGATAAAACGTACAGTCAA<br>R: CTGCATGCGATGTTTGAAG	VIC	KR558731
COC_10	tg	158-224	14	F: GCTCAGTCTACAAGCCTTCCA<br>R: GTGGATGACCAATCATGAGAA	NED	KR558732
COC_11	ag	101-129	14	F: ATCGCCGTGGAATTTGATAG<br>R: AATCGGTGGGTGGCTCTAC	6-FAM	KR558733
COC_13	ga	143-178	17	F: AGGGTTAGCTCTTTCGCCA<br>R: GGAGTTCGAAACGGCATAGA	6-FAM	KR558734
COC_18	ag	179-213	15	F:TGATTCATCAACCTAATTATTCATTCA<br>R: TGACTGATTTGTTTTCTCCTCC	NED	KR558735

Table S4 Description of eight microsatellite markers for *D. oliveri* developed for this study. Product size range and number of alleles based on 616 samples. Due to amplification problems OLI_01 was not included in the data analysis.

Marker name	Repeat motif	Product size range	# Alleles	Primer sequences	Fluorescent label	Genbank accession
OLI_01	ca	127-193	26	F: AAATAAAACTTATATGTTGCCAAACTC<br>R: TGGTACTAAGTTCCTGGACAACA	PET	KR558736
OLI_05	ag	124-164	18	F: GGATCAATTGGAGCCATACG<br>R: TTTGCGACTCTCGTGTAGTGA	6’FAM	KR558737
OLI_06	tg	111-149	19	F: ACTGGTAGGCCGACTTAACG<br>R: AAAATAGGCCATCATTAGTTTGC	VIC	KR558738
OLI_14	tg	180-239	24	F: TGAAATAAACCTAGCATCGACA<br>R: TGTTAGCATGAAGACAGGGTT	VIC	KR558739
OLI_15	ct	243-282	18	F: TTCATCAATGGTGGAGTTGG<br>R: CTGGCCAACCAGAATGATCT	PET	KR558740
OLI_16	ac	195-241	18	F: TAGAGGAGGGAAAGGAAGGG<br>R: AGCTGCCCTTCAGTTACAGTAT	NED	KR558741
OLI_17	tg	181-234	23	F: ATCATTCCATGTGGCTTCCA<br>R: TTTGATACTGATCTAACCACTGTCTT	6’FAM	KR558742
OLI_19	ctt	101-163	18	F: TTGATCATTACCCTTATTAGCCTT<br>R: CAGGAAGAAATTACAACCCCA	VIC	KR558743

Table S5 Results of AMOVA analysis with five regions and 26 populations of *D. cochinchinensis*.

Source of variation	DF	Variance components	Percentage of variation	
**Among regions**	4	0.571	16.71	F_{CT} **= 0.17**
**Among populations within regions**	21	0.341	9.98	F_{SC} **= 0.12**
**Within populations**	1020	2.506	73.31	
**Total**	1045	3.418		F_{ST} **= 0.27**

Table S6 Results of AMOVA analysis with six regions and 23 populations of *D. oliveri*.

Source of variation	d.f.	Variance components	Percentage of variation	
**Among regions**	5	0.199	6.75	F_{CT}**=0.07**
**Among populations within regions**	17	0.197	6.68	F_{SC}**=0.07**
**Within populations**	902	2.557	86.58	
**Total**	929	2.953		F_{ST}**=0.13**

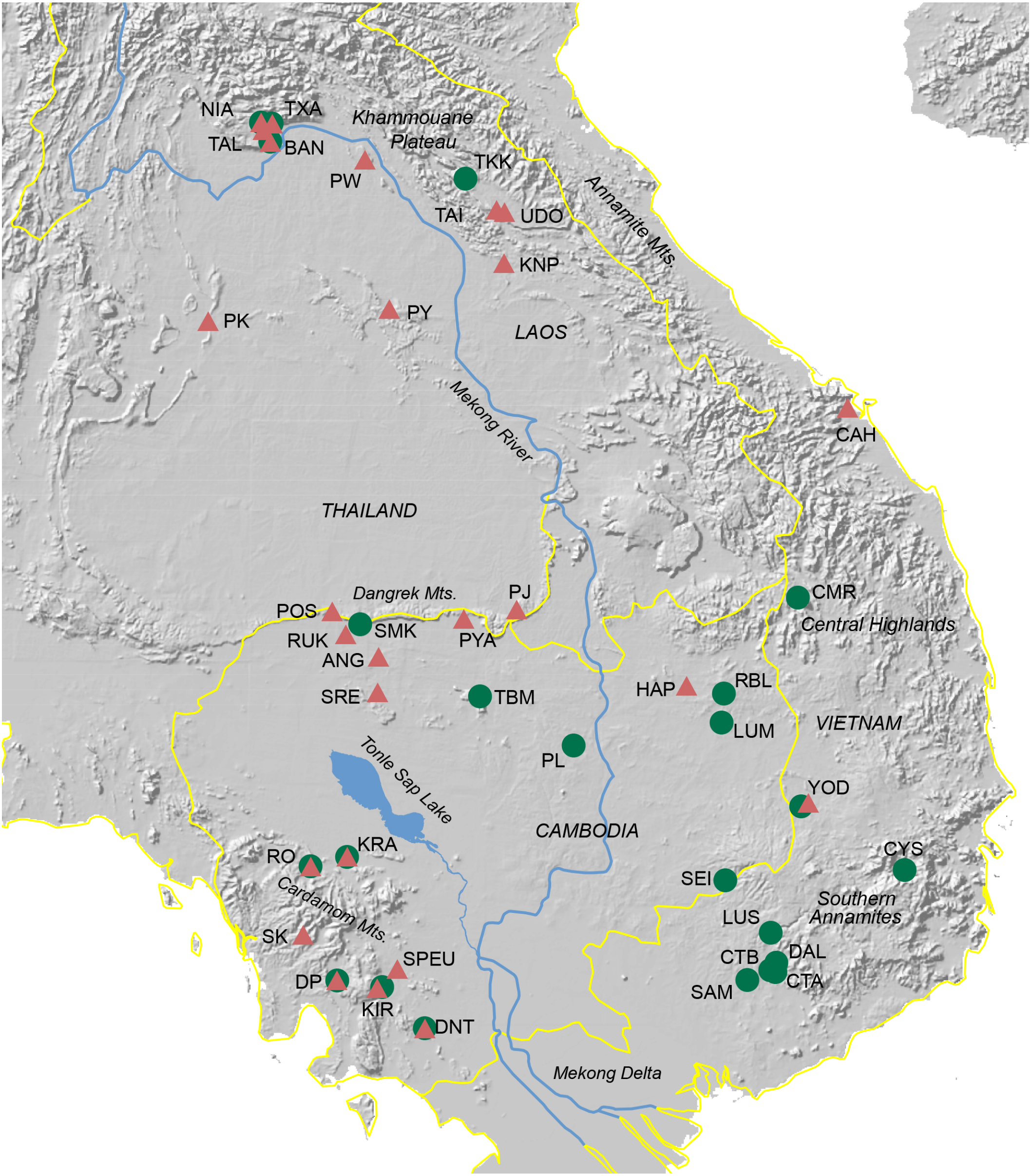

Figure S1 Map of sampled populations of *D. cochinchinensis* (red triangles) **and *D. oliveri*** (green dots)**.**

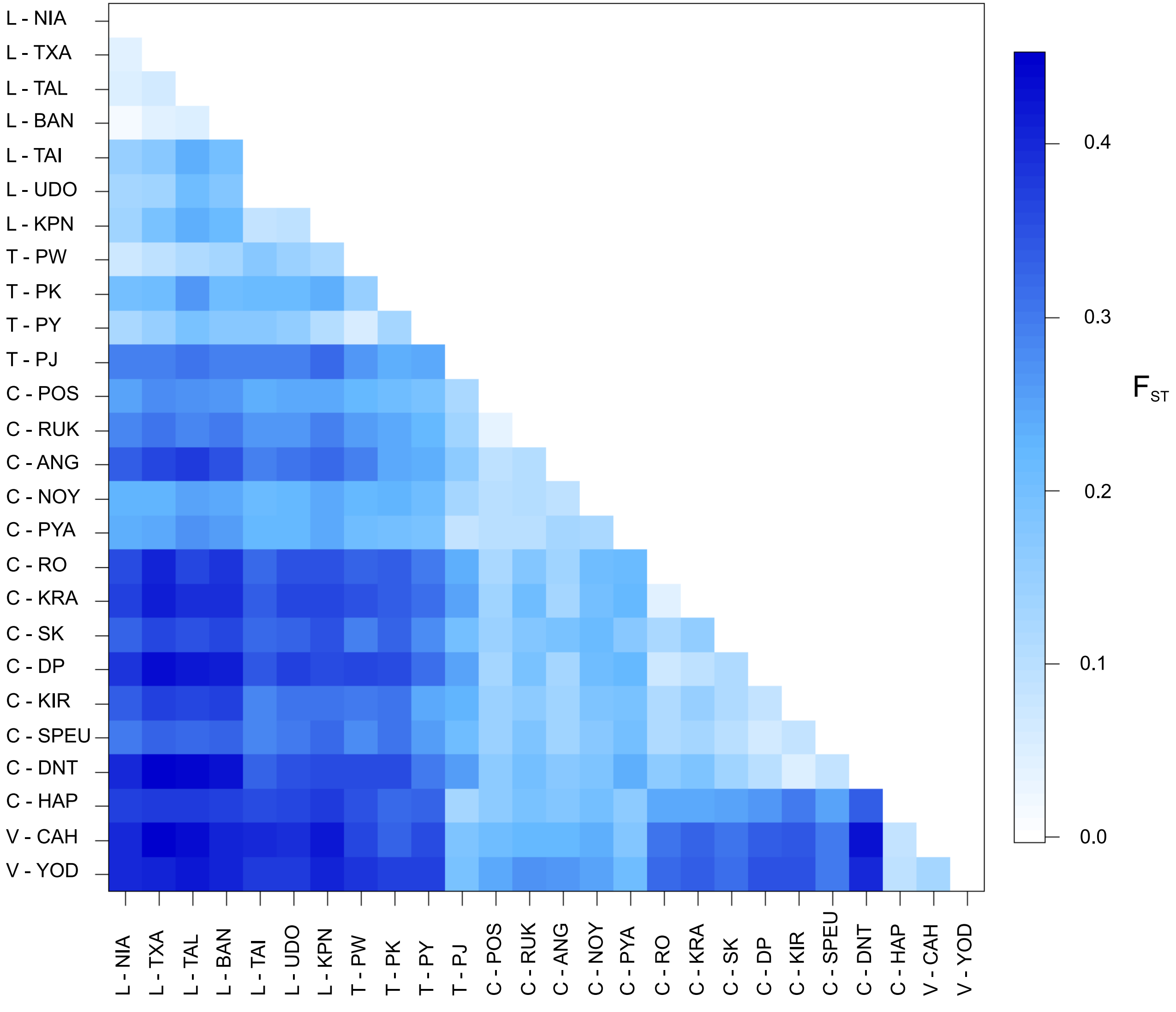

Figure S2 Pairwise *F_{ST}* values among all 26 populations of *D. cochinchinensis*. Darker color indicates stronger differentiation.

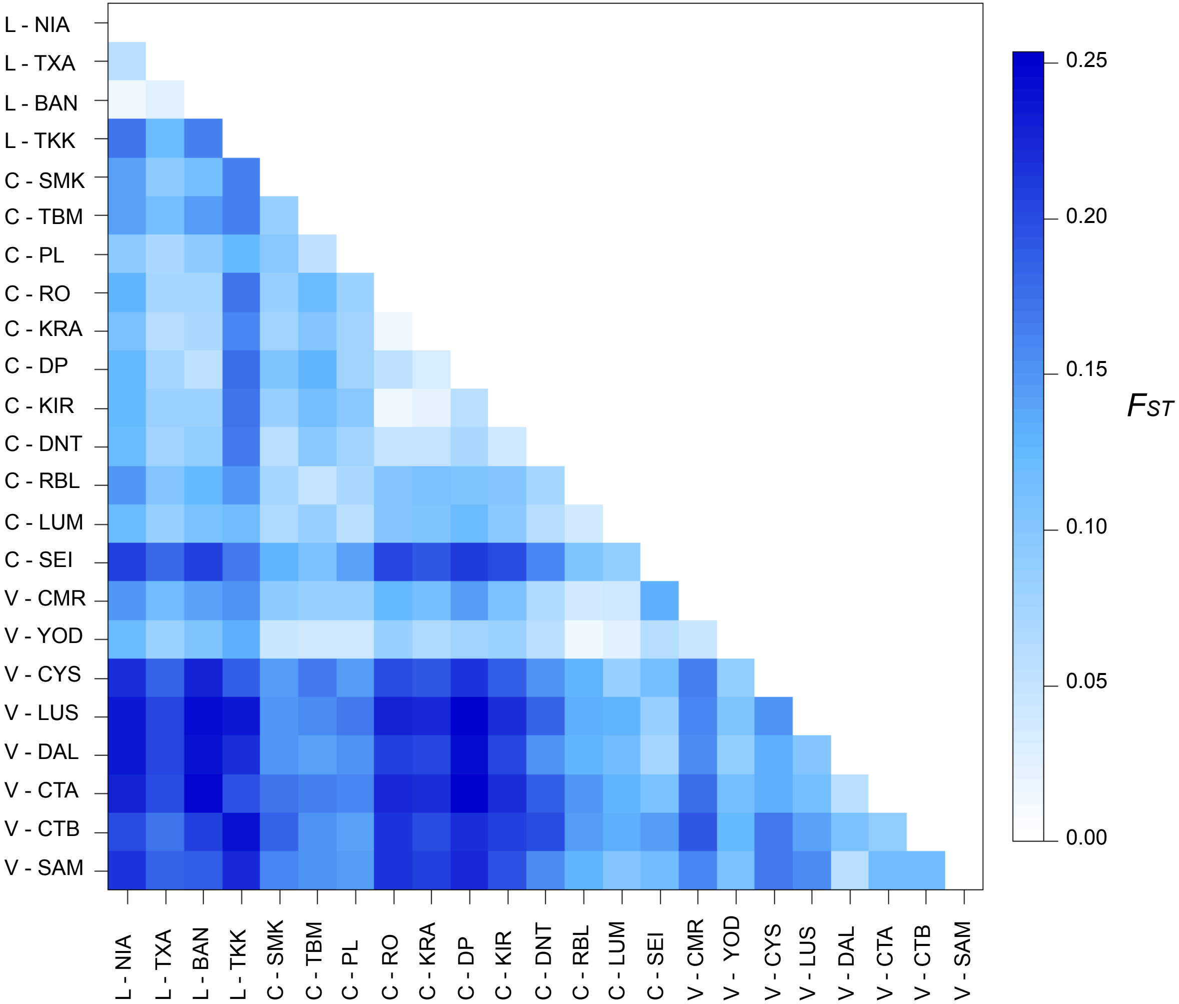

Figure S3 Pairwise *FST* values among all 23 populations of *D. oliveri*. Darker color indicates stronger differentiation

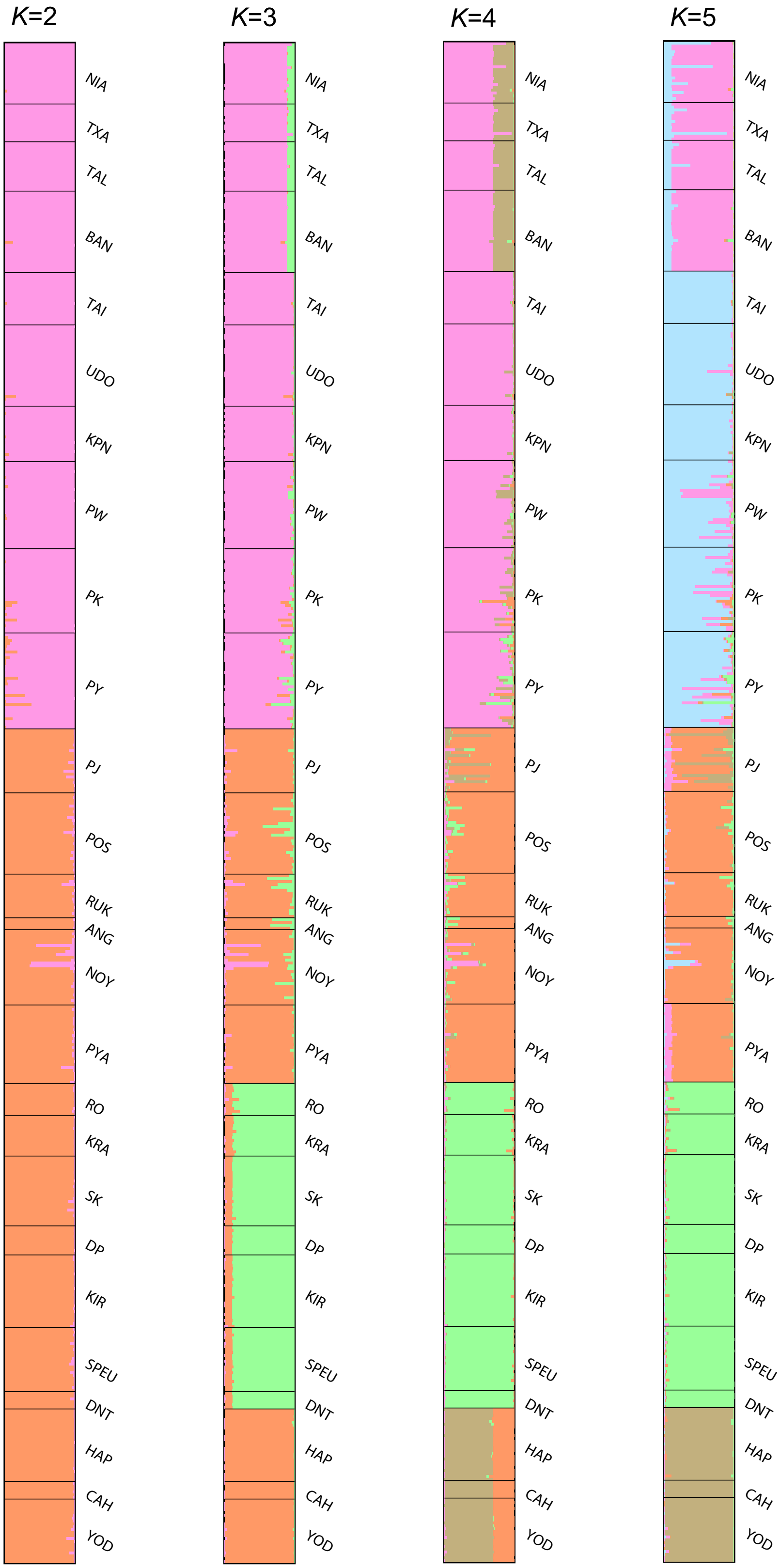

Figure S4 Structure results from K=2 to K=5 clusters for *D. cochinchinensis*. Each horizontal line shows the allocation of a single individual to the identified clusters, represented by different colors.

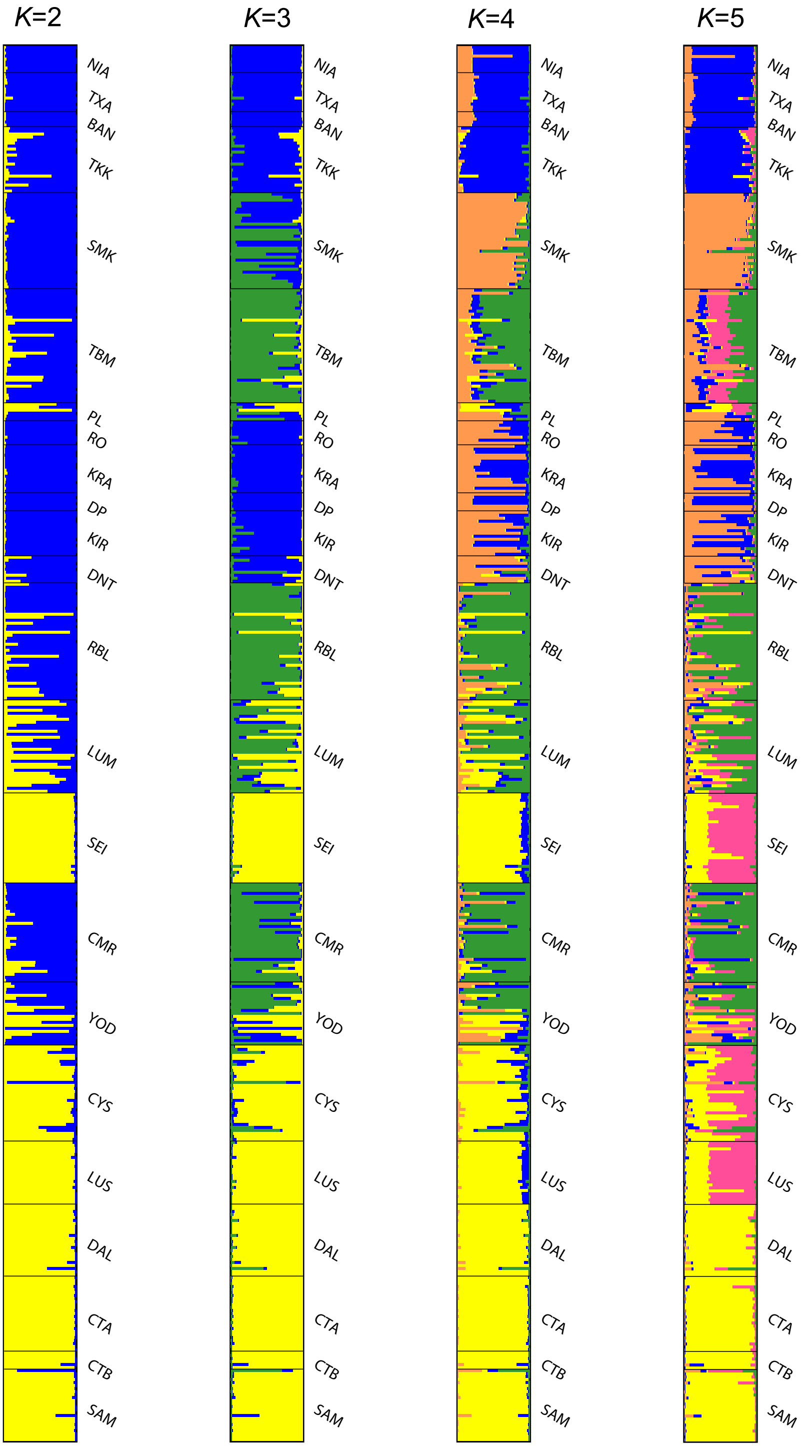

Figure S5 Structure results from *K*=2 to *K*=5 clusters for *D. oliveri*. Each horizontal line shows the allocation of a single individual to the identified clusters, represented by different colors.
